# Explaining Caregivers' Perceptions of Palliative Care Unmet Needs in Iranian Alzheimer's Patients: A Qualitative Study

**DOI:** 10.3389/fpsyg.2021.707913

**Published:** 2021-07-01

**Authors:** Hadis Ashrafizadeh, Mahin Gheibizadeh, Maryam Rassouli, Fatemeh Hajibabaee, Shahnaz Rostami

**Affiliations:** ^1^Student Research Committee, Nursing and Midwifery School, Ahvaz Jundishapur University of Medical Sciences, Ahvaz, Iran; ^2^Nursing Care Research Center in Chronic Diseases, Ahvaz Jundishapur University of Medical Sciences, Ahvaz, Iran; ^3^Cancer Research Center, Shahid Beheshti University of Medical Sciences, Tehran, Iran; ^4^School of Nursing and Midwifery, Tehran University of Medical Sciences, Tehran, Iran

**Keywords:** unmet need, palliative care, Alzheimers' disease, caregiver, Iran

## Abstract

**Introduction:** The needs of Alzheimer's patients are very complex and diverse and many of them are considered unmet needs. Understanding and responding to the unmet and complex needs of Alzheimer's patients can affect the quality of care. Therefore, the present study aimed to explain the perception of formal and informal caregivers of the unmet needs of Iranian Alzheimer's patients.

**Methods:** The present qualitative study employed a Directed Content Analysis approach and was conducted in Iran in 2020. This research was done through in-depth and semi-structured interviews with 19 qualified caregivers enrolled (11 informal caregivers and 8 formal caregivers) with the mean age of 46.05 ± 10.98 years in a purposive sampling method. Interviews continued until data saturation. Data analysis was performed simultaneously with data collection. After recording and transcribing, the data were analyzed using the Elo and Kyngas method based on the National Consensus Project framework (NCP). Data management was done with MAXQDA software. In order to achieve the accuracy and validity of the study, the criteria presented by Lincoln and Guba Credibility, Dependability, Confirmability, and Transformability were considered and used.

**Results:** The mean age of participants was 46.05 ± 10.98. Participants in this study acknowledged that Alzheimer's patients need comprehensive needs management with a holistic approach to increase quality of life. This theme based on the NCP framework consists of seven main categories including “Structure and processes of care,” “Physical aspects of care,” “Psychological and psychiatric aspects of care,” “Social aspects of care,” “Cultural aspects of care,” “Care of the patient nearing the end of life,” and “Ethical and legal aspects of care” with 16 subcategories.

**Conclusion:** The findings of this study provide a deep understanding of the unmet needs of Alzheimer's patients in Iran. Identifying the unmet needs of patients can pave the way for the treatment team to provide effective solutions to meet the needs and empower caregivers to provide comprehensive care for patients.

## Introduction

The growth of elderly population in the world has resulted in a considerable increase in the incidence of disabling disorders and cognitive problems (Farhadi et al., 2018). One of the most prevalent disorders accompanied by serious and progressive disability during old ages is Alzheimer's disease, which is the most common type of dementia (Craik and Salthouse, 2011; Ministry of Health, 2016). This disease is recognized by a variety of problems, such as amnesia, aphasia, apraxia, agnosia, and executive function disorder (American-Psychiatric-Association, 2013). The number of patients with Alzheimer's disease and other types of dementia in the world increased by nearly 117% from 1990 to 2016. Accordingly, the number of these patients increased from 20.2 million in 1990 to 40.8 million in 2016 (Alzheimer's-Association, 2018), and the prevalence of this disorder has been reported to be 3.6% in the Middle East (Adlimoghaddam et al., 2018). In Iran, it has been estimated that 700,000 elderly individuals suffer from Alzheimer's disease and that every 11.5 min, one person develops the disease (Salehi, 2011; Assocciation-Alzheimers-Iran, 2018).

Patients with Alzheimer's disease have various needs, such a way that their needs in the primary stage of the disease are quite different from those at the third and fourth (end) stages. Considering the incurable nature of Alzheimer's disease, it requires a long-term care approach in form of palliative care (Connor and Sepulveda Bermedo, 2020). Palliative care aims at meeting the needs of patients with incurable diseases, controlling pain, supporting patients' physical, mental, spiritual, and social needs, and improving patients' and their families' quality of life (Smith et al., 2012). Moreover, palliative care approach is compatible with the goals of person-centered dementia care. However, studies have revealed weak care standards among many patients with advanced Alzheimer's disease. In addition, no high-quality evidence is available for supporting palliative care approaches among these patients (Sampson, 2010).

Perceiving and meeting the needs of patients with Alzheimer's disease can directly affect care provision. Nonetheless, numerous studies have indicated that a large number of these patients' needs have remained unmet or have been met inappropriately (Cohen-Mansfield et al., 2015; Black et al., 2019; Mazurek et al., 2019; Sandman and Hofmann, 2019). Unmet needs could lead to a lower quality of life, higher levels of depression, worsening of neuropsychiatric symptoms, and increased disruptive behaviors (Hancock et al., 2006; Cohen-Mansfield et al., 2015). According to one study the major needs of elderly patients with Alzheimer's disease were the need for being heard, increase of knowledge level, and promotion of health (Bossen et al., 2009). Nevertheless, another research demonstrated that caring for patients with Alzheimer's disease was mainly focused on the physical dimension. In other words, these patients were provided with daily care services similar to other patients, while other dimensions of their needs were neglected by caregivers (Yektatalab et al., 2013). Hence, professional health care and family caregivers (formal and informal caregivers) are recommended to understand the needs of patients with Alzheimer's disease to be able to better find strategies to meet their needs.

Since perceptions and behaviors are shaped by culture, these needs and strategies for meeting them can be different depending on individual, cultural, and social differences (Zamanzadeh et al., 2014). In such countries as Iran, due to the existence of strong relationships among family members, patients with Alzheimer's disease are mainly cared by their own family members at home, also there is no codified plan to provide palliative care services, care is provided only in a limited number of centers, and patients do not receive the full range of essential care and training such as nursing care, physician consultation, and other care related to social, psychological, physical, or spiritual needs. Providing care, pain relief, and management of other symptoms in untreated patients by family members puts a lot of pressure on them (Rassouli and Sajjadi, 2016). Thus, the pressure imposed on patients and their family caregivers can result in a familial crisis. In this context, effective participation of formal caregivers in planning for a comprehensive care is of utmost importance, because they play a critical role in providing an appropriate environment as well as high-quality care for these patients (Yektatalab et al., 2013). Care dynamic in Alzheimer's disease requires taking individual, familial, caregiver, and social factors into account. Therefore, identification of formal and informal caregivers' viewpoints regarding patients' needs provides the ground for accurate evaluation of care services. Qualitative research is the best method for investigation of human phenomena and assessment of various perspectives, because human, social, cultural, and relational dimensions and values cannot be completely explored through quantitative approaches (LoBiondo-Wood and Haber, 2014). Moreover, qualitative studies can be used for in-depth description of unknown or less known phenomena from the perspective of individuals experiencing them in various cultures (Elo and Kyngäs, 2008). To the best of our knowledge, the available evidence has not well-explained the needs of patients with Alzheimer's disease. Hence, the present study aims to determine the perceptions of unmet palliative care needs among caregivers of patients suffering from Alzheimer's disease.

## Materials and Methods

### Study Design

This study was conducted using a directed content analysis approach in order to determine the perceptions of unmet palliative care needs among the caregivers of patients with Alzheimer's disease. This qualitative research aimed at development of a theoretical framework, because the existing knowledge regarding the intended phenomenon was not comprehensive and required further explanation (Elo and Kyngäs, 2008).

### Study Setting and Participants

The study participants included 11 informal caregivers of patients with Alzheimer's disease as well as eight formal caregivers, including physicians and nurses with the mean age of 46.05 ± 10.98 years. Eighty-four percent of the participants were female and the other 16% were male (Table 1). The participants were selected via purposeful sampling by referring to hospitals, nursing homes, and active branches of Alzheimer's Association in some provinces.

**Table 1:** Demographic characteristics of the participants in the study.

**Number**	**Participate**	**Age(year)**	**Gender**	**Marital status**	**Level of education**	**Job**	**Ratio with caregiver**	**Stage of patient**
1	Formal caregivers	58	Male	Married	Fellowship	Neurologist	Patient	Stage 1–4
2	Formal caregivers	38	Female	Married	Ph.D.	Doctor	Patient	Stage1–4
3	Formal caregivers	54	Female	Married	Fellowship	Faculty member	Patient	Stage1–4
4	Formal caregivers	37	Female	Married	Ph.D.	University professor	Patient	Stage1–4
5	Formal caregivers	38	Female	Married	Ph.D.	University professor	Patient	Stage1–4
6	Formal caregivers	47	Female	Married	Ph.D.	University professor	Patient	Stage1–4
7	Formal caregivers	43	Female	Married	Master sciences	University professor	Patient	Stage1–4
8	Formal caregivers	32	Female	Married	Master sciences	Nurse	Patient	Stage1–4
9	Informal caregivers	48	Female	Married	Diploma	Housewife	Father	Stage 3
10	Informal caregivers	71	Male	Married	Bachelor	Civil Engineer	Spouse	Stage 3
11	Informal caregivers	35	Female	Married	Bachelor	Housewife	Mother	Stage 2
12	Informal caregivers	52	Female	Single	Ph.D.	Nurse	Father	Stage 3
13	Informal caregivers	45	Female	Single	Master sciences	Teacher	Father	Stage 3
14	Informal caregivers	56	Female	Married	Illiterate	Housewife	Father	Stage 3
15	Informal caregivers	45	Female	Married	Diploma	Housewife	Father	Stage 4
16	Informal caregivers	65	Male	Married	Diploma	Housewife	Father	Stage 2
17	Informal caregivers	35	Female	Married	Diploma	Housewife	Mother	Stage 4
18	Informal caregivers	33	Female	Married	Illiterate	Housewife	Father	Stage 4
19	Informal caregivers	43	Female	Married	Illiterate	Housewife	Father	Stage 4

The inclusion criteria for the family caregivers were being able to speak Persian, aging at least 18 years, being the patient's first-degree relative, having been responsible for taking care of the patient for at least 6 months, not suffering from psychiatric disorders, and being willing to express one's experiences regarding taking care of a patient with Alzheimer's disease. Healthcare providers with at least 1 year of experience in provision of healthcare services for elderly individuals with Alzheimer's disease were also invited to take part in the research. The study was conducted in the participants' workplaces in order to investigate the intended phenomenon in the natural setting.

### Data Collection Procedure

The study data were collected using in-depth, semi-structured interviews from April 2020 to January 2021. Each interview lasted for 20–45 min. The interviews were continued until data saturation when the new data did not develop or modify the theory, change the existing categories, or provide suggestions for creating a new category (Gustavsson, 2007). After all, 22 interviews were conducted with 19 participants. The interviews with family caregivers were begun with the following question: “can you describe a normal day of taking care of your patient.” The interviews with formal caregivers were also begun with the following questions: “what services are usually demanded by patients and their families” and “which needs of patients and their families have not been met by service providers.” The main framework of interview questions is based on a conceptual framework such as different dimensions of care as well as end-of-life care. Then, probing questions, such as “can you explain more” and “can you provide an example,” were used for further investigations.

### Research Framework

Due to the variety of symptoms and the long and complicated process of Alzheimer's disease, management of this disease can impose huge expenditures on families (Harris, 2007). Hence, these patients require palliative care (van der Steen et al., 2014). This type of care was initially used for patients with cancer and HIV/AIDS. Afterwards, dementia, Alzheimer's disease, and other cognitive disorders were also added to the groups requiring palliative care (Batiste and Connor, 2017).

The conceptual framework of the present study was based on the National Consensus Project (NCP). In this project, palliative care has been defined as the activities focused on management of pain and other symptoms, evaluating and meeting caregivers' needs, and coordination of care, leading to physical, functional, mental, practical, and spiritual outcomes of a serious disease. Accordingly, palliative care is a personal and family-based attitude toward care, helps relieve people with serious diseases who suffer from the related stress and symptoms, and promotes the quality of life of patients and their families. The NCP has considered eight care dimensions for patients with restrictive and incurable disorders and has identified palliative care guidelines in these dimensions. These eight dimensions include the structure and process of care, physical dimensions of care, psychological dimensions of care, social dimensions of care, spiritual, religious, and existential dimensions of care, cultural dimensions of care, taking care of a dying patient, and ethical and legal dimensions of care (Ferrell et al., 2018).

### Data Analysis

The data were collected and analyzed simultaneously. Data management was done using the MAXQDA software (Kuckartz and Rädiker, 2019), and data analysis was done using the method proposed by Elo and Kyngas in preparation, organization, and reporting stages (Elo and Kyngäs, 2008; Elo et al., 2014). In doing so, each interview was read several times, so that the researcher gained an overall understanding of the content. Then, the meaning units were identified and the primary codes were extracted. After that, similar primary codes were classified in more comprehensive categories, and the main categories were extracted. Finally, all directed content analysis stages and results were abstracted and reported. Qualitative data analysis based on the method proposed by Elo and Kyngas has been presented in Table 2. In addition, an example of data analysis has been shown in Table 3.

**Table 2:** Qualitative data analysis process based on Elo Kyngas method.

Preparation stage	Select the analysis unit	After converting the interviews to text format, explicit content (same as the text of the interviews) and hidden content (non-verbal behavior of the participants) were analyzed and semantic units were identified.
	Find the logical connection of the data with the whole subject	The text of the interview was read several times by the researcher in order to gain a continuous and prolong engagement with the data.
Organizing stage	Create an analytical matrix	The structure and process of care, the physical aspect of care, the Psychological and psychiatric aspects of care, Care of the patient nearing the end of life, the social aspect of care, the cultural aspect of care, and the ethical-legal aspect of care were included as the main classes in the non-imposed matrix.
	Extract data from content based on categories	The main classes were formed based on conceptual and logical relationships with other classes, and finally the classes were identified based on the research framework.
	Grouping	The number of codes decreased by merging similar codes based on their differences and similarities into more general codes.
	Classification	The formed groups were classified based on their differences and similarities (merging similar groups).
	Abstraction	The revealed classes were placed in the main and primary classes of the analytical matrix.
Reporting stage	The sampling process, participants' characteristics, data collection, data analysis, and each of the main classes were reported in detail in the findings section.	

**Table 3:** An example of data analysis.

**Theme**	**categories**	**subcategories**	**Primary codes**	**quotation**
**Social aspect of care**	*Need for support on the part of the government and social systems*	*Need for support on the social systems*	**Establishment of supportive group groups** **Training through supportive group**	**We can use support groups to teach family and patient support (geriatrician)**.

### Rigor

In order to evaluate the qualitative data, use was made of the criteria proposed by Lincoln and Guba (Guba and Lincoln, 1994). Data credibility was ensured through prolonged engagement with the data (10 months), continuous observation, peer review, interaction among researchers, reviewing the interview transcripts by the participants, and combination of several methods (interview and observation). Considering transferability, the participants' features and experiences were described in details. In addition, the reliability of the data was ensured via accountability. In other words, research process, data collection and analysis, coding, and formation of categories were reviewed by an external observer who was familiar with qualitative studies. Finally, all research processes were recorded and presented extensively to ensure confirmability.

### Ethical Consideration

This study was approved by the Ethics Committee of Ahvaz Jundishapur University of Medical Sciences (IR.AJUMS.REC.1398.781). The ethical considerations in this study included the voluntary nature of participation in the research, provision of the participants with explanations about the study objectives, obtaining written informed consent forms, confidentiality of the participants' information, anonymity, the participants' right to withdraw from the study, and trustworthiness in using information and resources.

## Results

This study was conducted on 19 participants with the mean age of 46.05 ± 10.98 years. The participants' characteristics have been presented in Table 1.

The codes extracted from the interviews revealed one main theme; i.e., “needs management with a holistic approach,” which was in accordance with the NCP framework and consisted of seven main categories, namely “structure and process of care,” “Psychological and psychiatric aspects of care,” “Physical aspects of care,” “Care of the patient nearing the end of life,” “social aspects of care,” “cultural aspects of care,” and “ethical and legal aspects of care.” Detailed information about the main categories and subcategories has been provided in Table 4.

**Table 4:** Themes, main categories, and sub-categories resulting from the content-driven analysis of the interviews.

**Themes**	**Main categories**	**Sub-categories**
Needs management with a holistic approach	Structure and process of care	Need for development of diagnostic and screening services
		Need for specialized care services
		Need for home care services
		Need for hospice services
	Psychological and psychiatric aspects of care	Need for Psycho-emotional needs management
		Need for Psycho-cognitive needs management
	Physical aspects of care	Need for controlling progressive physical symptoms
		Need for controlling the secondary complications of treatment
	Care of the patient nearing the end of life	Need for comfort care
		Need for selecting a preferred place for death
	Social aspects of care	Need for support on the part of the government and social systems
		Need for empowerment of informal caregivers
	Cultural aspects of care	Need for destigmatization
		Need for cultural interventions
	Ethical and legal aspects of care	Need for ethical care
		Attention to patients' undeniable rights

### Needs Management With a Holistic Approach

In this study, the participants believed that patients with Alzheimer's disease required needs management with a holistic approach in order to enhance their quality of life. This was in accordance with the NCP framework and contained seven categories and 16 subcategories. This theme referred to the care structure and process for management of physical, psychological, and legal problems, resolution of cultural, ethical, and social issues, reception of extensive support from families and social and governmental institutions, and empowerment of family caregivers.

### Structure and Process of Care

This dimension included the assessment of palliative care, care program, and specific systems and processes for palliative care. This category consisted of four subcategories, namely “need for development of diagnostic and screening services,” “need for specialized care services,” “need for home care services,” and “need for hospice services.”

#### Need for Development of Diagnostic and Screening Services

Based on the study participants, diagnosis of Alzheimer's disease at the primary stages could impede the progression of the disease, which was considered one of the most effective strategies for preventing the incidence of the resultant disabilities. In this context, the participants referred to the differential diagnosis of Alzheimer's disease, prioritization of early diagnosis and identification of patients, and utilization of advanced tests for early screening of patients' problems including malnutrition and sleep disorders. For instance, one of the formal caregivers (geriatrician) maintained:

“*Patients have to be screened in different areas before the incidence of further problems; for example, regarding old age, malnutrition, cognitive disorders, and sleep*.”

#### Need for Specialized Care Services

“Need for specialized care services” was emphasized by the current study participants. This category referred to the required specialized care services, such as specialized rehabilitation services, empowerment of mental and cognitive status, psychiatric services, pharmacological treatments, visits by different specialists for controlling secondary problems, and provision of care services by day care, night care, and outpatient care centers. Considering the importance of pharmacotherapy, one of the formal caregivers (nurse) stated:

“*Pharmacotherapy is needed for patients in the primary phases and mild to moderate stages of the disease and reduces the speed of disease progression*.”

Regarding the importance of continuous visits by different specialists, one of the patients' spouse said:

“*In order to control the secondary problems, patients have to be visited by different specialists. In addition, patients have to be visited by a neurologist on a regular basis*.”

#### Need for Home Care Services

Home care, as a society-based care method, is one of the effective models in care provision that plays a key role in provision of comprehensive care services for patients with chronic diseases, including those suffering from Alzheimer's disease. In this disease, patients face challenges in doing their daily activities and as time goes by, a trained caregiver has to be with them at home or a nurse has to visit them at home on a regular basis. This category emphasized the necessity to design and develop home care services and to monitor the patient care process. In this respect, one of the patients' daughter mentioned:

“*We need centers for home care…A new structure should be designed…We do not have a hospitalized patient…They are not patients referred to health centers. We have an elderly patient who has a problem and is at home and is living in the society*.”

#### Need for Hospice Services

A large number of participants believed that due to the complex and variable conditions of patients with Alzheimer's disease who are in fact in the end stage of life, home care is not possible and they should be transferred to end-of-life care centers. In this regard, formal caregivers stated that the care services for patients at the end stage of life as well as their families had to be provided in form of palliative care in hospice centers. For instance, one of the formal caregivers (geriatrician) said:

“*The important point is that taking care of these patients at home is impossible in the long run, it's really impossible. It's illogical to expect families to take care of these patients until the last stages. There should be a place, so that these patients can be separated from their families in the last stages*.”

### Psychological and Psychiatric Aspects of Care

This dimension involved the psychological and psychiatric aspects of care in the field of Alzheimer's disease and consisted of two subcategories; i.e., “need for psycho-emotional needs management” and “need for psycho-cognitive needs management.”

#### Need for Psycho-Emotional Needs Management

All study participants believed that patients with Alzheimer's disease experienced various psycho-emotional disorders, including mood disorders, isolation, depression, anxiety, restlessness, behavioral disorders, aggression, confusion, and violent behaviors. These problems have to be identified in order to understand and respond to patients' needs, eventually providing the ground for providing them with comprehensive care services. In this respect, one of the patients' daughter stated:

“*For example, now he himself asks me why he is like this. He is depressed. He is sad. He says that he was not like this before. This is one of the features of Alzheimer's disease. They become depressed first and then, the depression causes them not to be able to eat or sleep and weakens their physical and mental statuses*.”

#### Need for Psycho-Cognitive Needs Management

The major complaints of the caregivers of elderly patients with Alzheimer's disease were related to cognitive issues, forgetting daily affairs, and inability to forge relationships with their families and other people. Since many cognitive deficiencies cannot be recovered in these patients, rehabilitation techniques are required to compensate for the deficiencies and to enhance the quality of life of patients and their families. In this context, one of the patients' daughter talked about her mother:

“*She got Alzheimer's disease little by little…Now, she just remembers her childhood memories, her old memories. Her mental and cognitive status even gets worse over time. She cannot build relationships, particularly with her family members…This is our problem. She cannot build relationships with us and she asks a lot of questions…You explain, but they ask the same questions*.”

### Physical Aspects of Care

In this dimension, evaluation of palliative care, care planning, and treatment of physical symptoms were described by focusing on comprehensive patient care. This category included two subcategories, namely “need for controlling progressive physical symptoms” and “need for controlling the secondary complications of treatment.”

#### Need for Controlling Progressive Physical Symptoms

The study participants stated that patients with Alzheimer's disease suffered from physical symptoms, including inability to do their daily activities (taking a bath and going to the toilet), loss of speaking ability, inability to stand up and walk independently, vulnerability to pain, urinary incontinence, weakness, cachexia, respiratory problems, panting, difficulty in swallowing, pressure ulcer, febrile infections, indifference to food, nausea, and vomiting, which had to be managed. In this regard, one of the patients' daughter said:

“*For example, my mother used to forget time and place at the beginning. We were most bothered by changes in her sleeping hours; she was awake all through the night, but slept throughout the day. Little by little, she was not able to do her daily chores, and someone had to be with her*.”

#### Need for Controlling the Secondary Complications of Treatment

Due to several pharmacological treatments, the disease complications are intensified in patients and the recovery process is slowed down. In the current study, some participants emphasized that unwanted complications occurred in patients with Alzheimer's disease because of the simultaneous consumption of different medications, suffering from different diseases at the same time, and reduction of body systems' functions. For instance, one of the patients' daughter mentioned:

“*After taking sleeping pills, my father had a fall and had a femoral head fracture. He didn't get well afterwards. We could interact with each other before that incident, but we can't now*.”

### Care of the Patient Nearing the End of Life

This dimension emphasized the common symptoms and conditions in the last days and weeks of life, and consisted of two subcategories; i.e., “need for comfort care” and “need for selecting a preferred place for death.”

#### Need for Comfort care

This type of care refers to improving the quality of life through relieving pain and other painful factors and providing practical, emotional, and spiritual support by focusing on patients' culture. In this study, a large number of caregivers believed that patients at the end stage of life needed different care services. These patients and their families needed tranquility, as well. One of the patients' daughter stated:

“*My father's condition was really complicated. He as well as all family members needed to be understood and supported*.”

Another patient's daughter also said:

“*When we can't do anything for our father, why should we bother him? Some painful measures should be disrupted, so that he will be comfortable*.”

#### Need for Selecting a Preferred Place for Death

One of the dimensions of end-of-life care was ensuring the patient's physical comfort at the time of death and selection of a preferred place for death. It also included preparing the family at this highly emotional and uncertain time. In this regard, one of the patients' daughter maintained:

“*When patients are at the end stages of life, they experience a terrible death process. The preferences and needs of the patients and their families have to be taken into account by the treatment team; the place in which the patient experiences a more comfortable death, not to be left alone*.”

### Social Aspects of Care

This dimension of palliative care referred to the evaluation and elimination of the social needs of the patients and their families. Based on the participants' statements, two subcategories were formed as follows: “need for support on the part of the government and social systems” and “need for empowerment of informal caregivers.”

#### Need for Support on the Part of the Government and Social Systems

Support on the part of social systems and entrusting some responsibilities to a group of individuals who can provide those in need with emotional protection and support resources were among the needs extracted from the present study results. Almost all study participants mentioned that inappropriateness of the price of the required medications and equipment to the patients' financial ability, lack of insurance coverage, and high costs of screening and diagnostic tests had caused numerous challenges for the patients and their families. Therefore, in order to afford the costs of life, medications, and treatments, patients need to be supported by the government, social institutions, support groups, peers, active NGOs, and charities. These support resources help individuals feel cared for, loved, and valued, thereby increasing their self-esteem. Considering the importance of NGOs, one of the formal caregivers (physician) maintained:

“*NGOs should be asked for help in this area. In Iran, we don't have active NGOs in the field of Alzheimer's disease. If we can establish such centers; this was started in some parts of the country, but was not completed*.”

#### Need for Empowerment of Informal Caregivers

This part emphasized the necessity of informational, mental, and emotional support for caregivers, which was in line with empowerment of informal caregivers for facing problems and managing care. The study participants believed that entrusting care to the informal caregivers who did not have the required knowledge and experience for taking care of patients with Alzheimer's disease could result in the quick progression of the disease. Hence, they stated that families had to be provided with information and support in order to provide appropriate care services, manage and control the disease complications, develop communication skills with patients, and manage the crisis and stress. Regarding the caregivers' inability to provide proper care, one of the informal caregivers who was responsible for taking care of one's father mentioned:

“*The family caregiver didn't know what to do with the patient's constipation, how to prevent pressure ulcer in the patient with Alzheimer's disease, what to do with the patient's dry joints. S/he didn't even know how to provide the intended position*.”

### Cultural Aspects of Care

This dimension dealt with the effective cultural factors in the provision of palliative care and provision of patients and their families with services. It consisted of two subcategories, namely “need for destigmatization” and “need for cultural interventions.”

#### Need for Destigmatization

Stigma has been defined as the outcomes associated with unawareness, prejudgment, and discrimination about an issue. Generally, stigma causes families to suffer from emotional experiences, such as disrespect, indifference, and discrimination. Besides, fear from stigma results in secrecy. Some participants in this study mentioned that they tried to hide their patients since they were afraid of being teased by others. This feeling of shamefulness due to the patient's repeated questions and abnormal behaviors as well as others' judgments could be attributed to the association of this disease with mental disorders. In this respect, one of the informal caregivers stated:

“*Others' behavior is what makes us sad…For example, when I took my mother to other places, others said that it was very hard and asked us how we took care of the issue and how we tolerated her…Shouldn't we tolerate her? She is our mother; she has done everything for us for a lifetime. Now, it's our duty*.”

#### Need for Cultural Interventions

In this part, the participants remembered the cultural items, which were less taken into consideration in the society. In fact, culture is a concept, which is taught and transferred to individuals through socialization. In this dimension, emphasis was put on training through the mass media in order to increase awareness about aging and the associated challenges as well as modification of the society's attitude toward Alzheimer's disease (destigmatization). Regarding the importance of training and Culturalization, one of the formal caregivers (physician) maintained:

“*Culturalization should be done. This does not occur simply. It should be taught through the radio, TV, and mass media*.”

### Ethical and Legal Aspects of Care

The contents of this dimension included planning for advanced care services, decision-making for sensitive issues, and ethical and legal considerations with a focus on the necessities and ethical processes for supporting patients' independence. This dimension consisted of two subcategories; i.e., “need for ethical care” and “attention to patients' undeniable rights.”

#### Need for Ethical Care

One of the axes of the patients' rights charter is the respect for patients' and their families' right for selecting and making decision about receiving healthcare services. For instance, the participants expressed that patients and their families had to be involved in making decisions about their treatment. In addition, families had to give their patients the right to maintain their independence in doing their daily activities. In this context, one of the informal caregivers (a girl taking care of her father) said:

“*During his operation, for example, I said a hundred times that he had Alzheimer's disease, but it seemed as if I was talking to no one. Or during his femur fracture operation, no one asked my opinion as the caregiver, as the nurse. They do not explain what they are going to do for the patient. Nobody asks about the patient's history and conditions*.”

#### Attention to Patients' Undeniable Rights

This category referred to decision-making about patients' legal issues, including management of their possessions, writing a will, and selecting a guardian and a main caregiver. In this regard, some participants mentioned the legal issues occurred for patients with Alzheimer's disease, because these patients have judgments and decision-making disorders and, consequently, are prone to financial abuse on the part of their acquaintances. For instance, one of the formal caregivers (geriatrician) maintained:

“*Patients face increasing problems in keeping their daily financial accounts. Therefore, elderly people should be provided with the related information. There may be resistance on the part of elderly individuals or their acquaintances; e.g., writing a will, announcing a person as the guardian at the severe stage of the disease. All these should be taught and individuals should decide who to be the main caregiver*.”

## Discussion

Identification of unmet palliative care needs among patients with Alzheimer's disease and consideration of the complexity of these needs can help healthcare providers to provide them with the care services appropriated to their needs. The present study aimed to determine the perceptions of unmet palliative care needs among the caregivers of patients with Alzheimer's disease. The codes extracted from the interviews resulted in the emergence of the theme “Structure and processes of care,” “Physical aspects of care,” “Psychological and psychiatric aspects of care,” “ Social aspects of care,” “ Cultural aspects of care,” “Care of the patient nearing the end of life,” and “Ethical and legal aspects of care” with 16 subcategories.

As mentioned above, the theme extracted in the present study was management of patients with Alzheimer's disease using a holistic approach Comprehensive management refers to a value-based decision-making framework, which integrates all planning dimensions and is one of the major principles of palliative care. The results of numerous studies have emphasized the necessity to comprehensively manage the behavioral problems, personal, care, and emotional activities, and social needs of patients with Alzheimer's disease (Cadieux et al., 2013; Sinvani et al., 2018; Zucchella et al., 2018). The findings of a prior research indicated that the Severe Behavior Response Teams (SBRT) model was used for patients with dementia in Australia. This model puts emphasis on out-of-home services and the patient's living place. In this model, services are provided by a multidisciplinary team in response to crises. Interventions are initially focused on the evaluation and management of behaviors and are completed by training and supporting caregivers (Macfarlane and Cunningham, 2017). In Iran, some programs have been developed and started for dealing with Alzheimer's disease. However, there is no comprehensive approach for management of the needs of these patients. Moreover, lack of care centers and home care systems for taking care of patients with Alzheimer's disease are considered the main obstacles against comprehensive management of the disease in Iran. Hence, this approach is recommended to be taken into account (Yektatalab et al., 2013).

“Structure and process of care” was among the main categories extracted from the interviews. Provision of comprehensive care services requires structures to take the responsibility of providing a part of the services. This construct emphasizes screening and early diagnosis, and indicates that hospitalization of patients at the specialized level depends on diagnosis at lower levels and treatment, pharmacological, and rehabilitation services are provided on the basis of patients' needs. Home care services, as society-based services, were also included in this category. In case patients do not have complicated needs, home care services can be provided, which is of interest to a large number of patients and their caregivers. This type of care refers to reception of a wide range of care services appropriated to patients' needs. There is limited evidence regarding society-based home care services for supporting patients with dementia. Dawson et al. (2015) demonstrated that home care services were accompanied by desirable outcomes in case they were provided on time and were responsive, flexible, and appropriated to individual needs (Dawson et al., 2015). This category also referred to hospice centers for patients with Alzheimer's disease at the end stage of life. Hospice centers provide high-quality spiritual, mental, social, and physical services for patients and their caregivers. The findings of the research by Mitchell et al. (2007) revealed that hospice service providers could respond to the unique challenges of end-of-life care for patients suffering from dementia (Mitchell et al., 2007). The studies performed in Iran have also emphasized the need for establishment of hospice centers for provision of care services at the end stages of life. Despite the increasing demands for care services at the end stages of life due to the increasing incidence of chronic and life-threatening diseases in Iran, there are no hospice centers for the patients and families who require optimum care services (Azami-Aghdash et al., 2015). The most important reasons for the lack of hospice centers in Iran include the lack of a proper ground, lack of expert human workforce, lack of comprehensive care programs, lack of appropriate guidelines, lack of policies, lack of free access to opioids, financial problems, economic problems, and cultural differences (Assocciation-Alzheimers-Iran, 2018; Zarea et al., 2020).

“Psychological and psychiatric aspects of care” and “physical aspects of care” were two other categories extracted in the current investigation. In order to organize integrated healthcare services for meeting the needs of patients with Alzheimer's disease, the variety of symptoms and needs in this population should be taken into consideration (Farmer et al., 2016; Commisso et al., 2017). In fact, designing person-centered rather than disease-focused care services is only possible through perception and prioritization of patients' symptoms and problems (Curnow et al., 2019). The Behavioral and Psychological Symptoms of Dementia (BPSD) include a wide range of physical, emotional, psychological, and behavioral symptoms (Tible et al., 2017) whose management and treatment are challenging due to the complex etiopathogenesis of the symptoms as well as multiple complications. Management of BPSD involves an accurate diagnostic investigation, exploration of the causes of Alzheimer's disease, and elimination of other causes such as delusion related to drugs consumption, simultaneous treatment of other physical diseases, effective control of pain or infections, and providing patients and their families with psychosocial therapies (Hersch and Falzgraf, 2007; Tible et al., 2017). In Iran, management of BPSD symptoms is mainly focused on pharmacological interventions. Expert advice and guidance, prefer non-pharmacological interventions as a first-line approach although the evidence for most non-pharmacological strategies is weak, their effectiveness is confirmed by long-term clinical experience. Medication for BPSD is offered frequently, but carries the risk of serious side effects (Savaskan et al., 2014; Deuschl and Maier, 2016). Nonetheless, patients suffering from Alzheimer's disease are vulnerable to the side effects of medications. Hence, non-pharmacological interventions are recommended to be performed for better management of physical and psychological symptoms amongst these patients.

“Care of the patient nearing the end of life” was the fourth dimension extracted in the present research. Taking care of dying patients is a huge challenge, which is done in a challenging environment. In this dimension, patient care involved “comfort care” and “selection of a preferred place for death,” which was provided comprehensively with a focus on the patient's body and mind (Gillan et al., 2014). Generally, end-of-life care aims at providing the ground for a comfortable death, maintaining balance in patients' daily lives, and consoling their families (Iranmanesh et al., 2010). In fact, end-of-life care is a key component of taking care of elderly individuals. Nevertheless, evidence has indicated that individuals aging above 85 years had little access to specialized end-of-life care services (Hunt et al., 2014). Furthermore, the findings of some studies have shown that pain control through the use of analgesics, management of symptoms, palliative care, and Do Not Resuscitate (DNR) and Do Not Hospitalize (DNH) guidelines were carried out less among patients with Alzheimer's disease in comparison to other patients (Mitchell et al., 2004; Sampson et al., 2006).

“Social aspects of care” was another main category in the current research. One of the subcategories of this dimension was the “need for support on the part of the government and social systems.” Accordingly, patients with Alzheimer's disease required formal care support or access to care services to meet their needs (Ball et al., 2015; Smith et al., 2015; Hynes et al., 2016). Creating and taking part in support groups formally, informally, and voluntarily could be a proper measure for gaining knowledge about coping strategies as well as emotional and informational support (Rosa et al., 2010). Based on the reports provided by Prince (2004), patients with Alzheimer's disease in Iran received lower social support compared to those living in developed countries (Prince, 2004). These results were in agreement with those of the present investigation (Górska et al., 2018; Yang et al., 2020). In the Netherlands, for instance, a Meeting Centers Support Program (MCSP) is used as a supportive approach for patients with mild to moderate Alzheimer's disease who live in the society as well as for their caregivers. This approach is a combination of recreational and psychotherapeutic activities for patients, psychotherapy groups for caregivers, social activities for patients and caregivers, and holding regular sessions in treatment centers (Dröes et al., 2000; Farina et al., 2006). Another subcategory of this dimension was the “need for empowerment of informal caregivers.” The results of a previous research indicated that in case caretaking was carried out continuously by caregivers, lack of support programs on the part of the government, society, and family members could increase the burden of the caretaking responsibility and the probability of fatigue in the main caregivers, which might lead to misbehavior toward patients with Alzheimer's disease (Mohamadi et al., 2008). In another study, emphasis was put on emotional, mental, and informational support for caregivers. In addition, empowerment of caregivers for facing the problems associated with taking care of elderly patients as well as management of family caretaking were considered important (Mohammadi, 2008). In spite of the need for social support, there are no support programs, including financial, mental, and social support programs, on the part of government organizations, peers, families, and the society for patients with Alzheimer's disease in Iran.

Another category extracted in the present research was “cultural aspects of care,” one of whose subcategories was the “need for destigmatization.” Generally, diagnosis of Alzheimer's disease, similar to mental disorders, can be accompanied by considerable stigma, which may be attributed to the cultural beliefs about the etiology of the disease as well as to the patients' unsocial behaviors that can result from cognitive disorders (Mukadam and Livingston, 2012; Navab et al., 2019). In this study, the caregivers' experiences indicated that they were afraid of being teased by their acquaintances as well as by the society and, as a result, attempted to hide the patients. In general, people living in collectivist cultures like the Iranian culture pay great attention to their relationships with other individuals. When patients are not able to build effective relationships with their caregivers and other individuals, their membership in the society will be questioned. Hence, these patients need to be accepted by their family members, the society, and the healthcare system (Yektatalab et al., 2013). Therefore, it is necessary to carry out cultural interventions, including training through the mass media, in order to improve the society's attitude toward these patients and their families. The results of a study explained that stigma among patients with Alzheimer's disease could be reduced via Culturalization, training, protesting against the existing inequities, and forging relationships with these patients (Mukadam and Livingston, 2012). Edney also disclosed that mass media could exert a great impact on people's belief systems. Thus, showing mental disorders and Alzheimer's disease was reported to be effective in the perception of the disease (Edney, 2012).

The last category extracted in the current study was the “ethical and legal dimensions of care.” Since Alzheimer's disease has a progressive nature and patients' abilities are considerably decreased at each stage, adhering to ethical and legal considerations is essential in each stage of the disease. Khan Ahmadi et al. (2015) mentioned the ethical considerations related to patients with Alzheimer's disease in form of independence, care, participation, self-fulfillment, and dignity principles in three sections (care, treatment, and research) at the three stages of the disease (beginning, middle, and final) (Khan Ahmadi et al., 2015). In spite of the rich Iranian culture that values respect toward elderly people, the ethical and legal principles for this group are not taken into account nowadays. In some countries like England and Wales (2005), however, the Mental Capacity Act has been approved and executed for solving the ethical and legal problems of patients with Alzheimer's disease. This program provides these patients with a framework to decide for themselves and to choose representatives for making decisions about their behaviors. This includes basic decisions about care, treatment, financial issues, and daily affairs (Kritika Samsi, 2012). Unfortunately, these issues are not common in Iran and even a power of attorney can be revoked in case insanity is diagnosed in any of the parties. This is in fact a legal gap, which requires quick modification.

## Study Limitations

Even though qualitative studies show the participants' in-depth experiences, they may suffer from the non-generalizability of the results. One of the limitations of the current study was that most of the participants were female. Another significant study limitation was related to the selection of caregivers in the research setting, which was faced with problems due to the COVID-19 pandemic.

## Conclusion

The study findings demonstrated that patients with Alzheimer's disease suffered from numerous physical, mental, emotional, and cognitive problems. In addition, disease symptoms and complications, lack of a holistic and comprehensive care system, social stigma, and lack of acceptance by the society, cultural, ethical, and legal issues associated with the disease, lack of support resources, and the economic burden of the disease caused patients and their families to face various challenges. Thus, paying attention to patients' unmet needs could result in beneficial effects for both patients and their informal caregivers. These needs can be perceived by determining the experiences of formal and informal caregivers. This issue is of particular importance in Iran, which is faced with the aging phenomenon. Overall, identification of patients' unmet needs can help health systems to take effective measures for meeting those needs and strengthening caregivers for providing patients with comprehensive care services.

## Data Availability Statement

The original contributions presented in the study are included in the article/supplementary material, further inquiries can be directed to the corresponding author/s.

## Ethics Statement

The studies involving human participants were reviewed and approved by the Ethics Committee of Ahvaz Jundishapur University of Medical Sciences (IR.AJUMS.REC.1398.781). The ethical considerations in this study included the voluntary nature of participation in the research, provision of the participants with explanations about the study objectives, obtaining written informed consent forms, the confidentiality of the participants' information, anonymity, the participants' right to withdraw from the study, and trustworthiness in using information and resources. The patients/participants provided their written informed consent to participate in this study. Written informed consent was obtained from the individual(s) for the publication of any potentially identifiable images or data included in this article.

## Author Contributions

MG, HA, and MR in critical revisions for important intellectual content and administrative/technical support and supervised the work. All authors involved in the study conception, design and contributed to the data collection, and analysis.

## Conflict of Interest

The authors declare that the research was conducted in the absence of any commercial or financial relationships that could be construed as a potential conflict of interest.
